# A novel mutation of *KCNJ1* identified in an affected child with nephrolithiasis

**DOI:** 10.1186/s12882-022-02783-x

**Published:** 2022-06-27

**Authors:** Saisai Yang, Guanghui Yao, Xin Chen, Huirong Shi, Chihhong Lou, Shumin Ren, Zhihui Jiao, Cong Wang, Xiangdong Kong, Qinghua Wu

**Affiliations:** 1 Department of Obstetrics and Gynecology, Center of Genetics and Prenatal Diagnosis, The First Affiliated Hospital of Zhengzhou University, Zhengzhou, 450052 Henan China; 2 Gene Editing and Viral Vector Core, City of Hope Medical Center, Duarte, Los Angeles, CA 91006 USA

**Keywords:** Nephrolithiasis, Next generation sequencing, *KCNJ1*, Bartter syndrome

## Abstract

Nephrolithiasis is not common in children, but the incidence is gradually increased in these years. Urinary tract malformations, urinary infection, dietary habits, geographic region and genetic factor are involved in the etiology of nephrolithiasis. For the affected child, it is especially important to elucidate the etiology, which may provide an accurate diagnosis, a personalized therapy and effective follow-up strategy. Here to seek the etiology of a ten-year-old boy incidentally found with nephrolithiasis, next generation sequencing (NGS) including a panel with 248 genes involved in hereditary kidney diseases was performed for the boy and identified two mutations of *KCNJ1*, c.89G > A (p.C30Y) and c.65G > T (p.R22M), and the later was a novel missense mutation originated from his father. The child was confirmed with type II Bartter syndrome (BS) caused by *KCNJ1* mutations. Our study suggests that BS may be difficult to get diagnosed at an early stage based on clinical manifestations or biochemical laboratory tests, and NGS is an efficient way to determine the etiology and provide further treatment and guide fertility counseling for the affected family.

## Introduction

Nephrolithiasis is not common in children compared to adults, but the incidence of pediatric nephrolithiasis is gradually increased in these years [1]. Urinary tract malformations, urinary infection, dietary habits, geographic region and genetic factor are involved in the etiology of nephrolithiasis [2, 3]. There are at least 30 genes shown to cause monogenic forms of nephrocalcinosis or nephrolithiasis in autosomal-dominant, autosomal-recessive, or X-linked transmission patterns [4]. For the affected child, it is especially important to detect the exact causative mutation of monogenic disease and know the etiology, which may provide an accurate diagnosis, personalized therapy and effective follow-up strategy.

In the present study, we described the clinic features of one 10-year old child who was incidentally found with nephrolithiasis and detected with the genetic mutation, which suggests that NGS is very important and efficient to identify the etiology of nephrolithiasis, which may not be easily diagnosed by manifestation or chemical laboratory tests.

## Materials and methods

### Enrollment of human subjects

This study complies with the Declaration of Helsinki and has been approved by the Ethics Committee of the First Affiliated Hospital of Zhengzhou University (ID: KS-2018-KY-36). Written informed consent has been obtained from the patient’s guardian for the release of the case report and any accompanying images. Copies with written consent are available for editorial review of this journal.

### Next generation sequencing (NGS) and sanger sequencing

Targeted-NGS using inherited kidney disease panel including 248 disease-causing genes was performed by a commercial company (MyGenostics, Inc., Beijing, China). To validate the variants screened by NGS, the related fragments were performed PCR amplification for the proband and his parents, and the primers of the fragments were listed in Table 1. PCR products were bi-directionally sequenced using an ABI 3730XL sequencer (Applied Biosystems, Foster City, CA) in the Center of Genetics and Prenatal Diagnosis of the First Affiliated Hospital of Zhengzhou University.

**Table 1:** Primers used for pcr amplification of *kcnj1*

Primer	Primer sequence	Location	Mutation site	Fragment size (bp)	SIFT	Polyphen-2	MutationTaster	REF
F	CGCTACTGCATACCACAGGAG	Exon 4	c.89G > A	400	0	0.999	1	Schulte et al. 1999
R	TGCCAAATGATTAGTAACCCAG		c.65G > T		0	0.998	1	Novel

### Bioinformatics analysis

The harmful prediction was analyzed according to the scoring conditions using three kinds of software including SIFT, PolyPhen-2 and MutationTaster. The pathogenicity of the mutation site was annotated according to the American College of Medical Genetics and Genomics (ACMG) guidelines.

### Conservation analysis and molecular modeling of the protein encoded by *KCNJ1*

To evaluate the evolutionary conservation of the mutated site, the apical potassium inwardly-rectifying channel (ROMK) encoded by *KCNJ1* from five animal species from fishes to mammals, including human (*Homo sapiens*: NP_722451.1), zebrafish (*Danio rerio*: NP_957329.1), mouse (*Mus musculus*: NP_062633.1), cattle (*Bos taurus*: NP_001179136.1), rat (*Rattus norvegicus*: P35560–2) were analyzed.

The initial mutant variant structures for ROMK (Residues1–372) were constructed using the automated protein-homology modeling server SWISS-MODEL, using the protein structure of an Inward rectifier potassium channel as a structural template (PDB: 3SPG). PROCHECK was employed to estimate the quality of our models. There are 98.6% residues located in the ‘core’ and ‘allowed’ regions, 1.3% in the ‘general’ region and only 0% in the ‘disallowed’ region. In the computational structure, 99.8% of the bond lengths for the main-chain residues and 99% of the bond angles for the main-chain residues are within the allowed limits. The sequence identity was 47.42%. Analysis of the 3-D structure of the proteins was carried out using Pymol.

## Results

### Clinical examinations

**Fig. 1:**
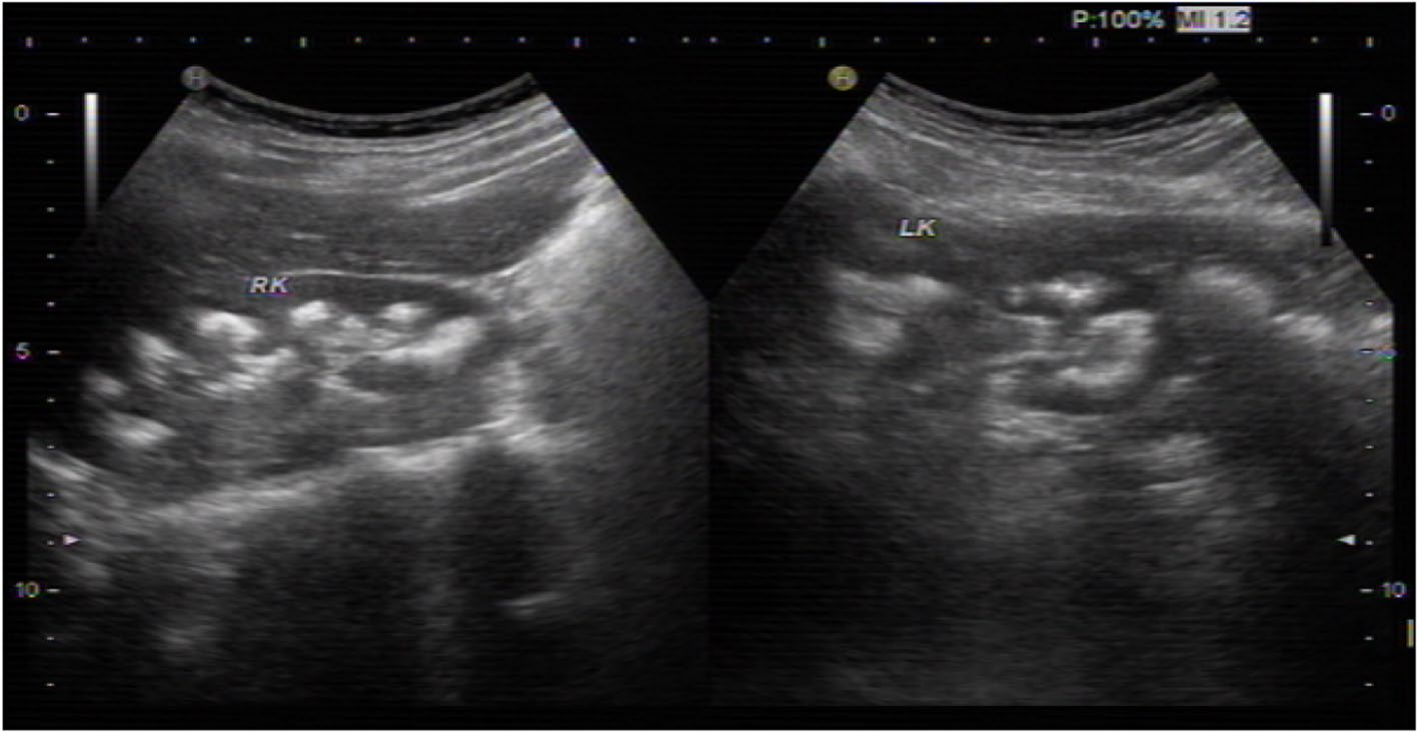
The urinary ultrasound showed enhanced echo of bilateral renal collecting system

The proband was 10-year old, and the weight was 28 kg (−1standard deviation [SD] for age), with the height 130 cm (− 2 SD). The other general physical examinations of the proband were normal, with a normal blood pressure of 123/65 mmHg. Biochemical laboratory tests showed blood routine, electrolyte, liver function, renal function, urinary routine were normal. The urinary ultrasound showed enhanced echo of bilateral renal collecting system, several echogenic foci with posterior shadow, the largest one on the left kidney 8 mm × 6 mm, on the right side 9 mm × 6 mm (Fig. 1), the blood perfusion was normal, and multiple high echo spots in the prostate with posterior shadow, the largest one 6.4 mm × 4.6 mm. Abdominal computed tomography (CT) showed multiple high density shadows in bilateral renal medulla and left high density nodules of urethra prostate. Glomerular filtration rate (GFR) in the left kidney was 34.07 ml/min shown by SPECT-CT, indicating mild impairment of left renal function (normal range: 40–50 ml/min), and GFR in the right kidney was 51.11 ml/min. Traced back to the previous history of the proband, he was born prematurely at 31 weeks of gestation with low birth weights about 1.7 kg following polyhydramnios. No obvious abnormality was seen until 4 years ago, and he presented intermittent cramps, fatigue and muscle weakness and these symptoms were released after taking oral potassium chloride.

### Mutation analysis

**Fig. 2:**
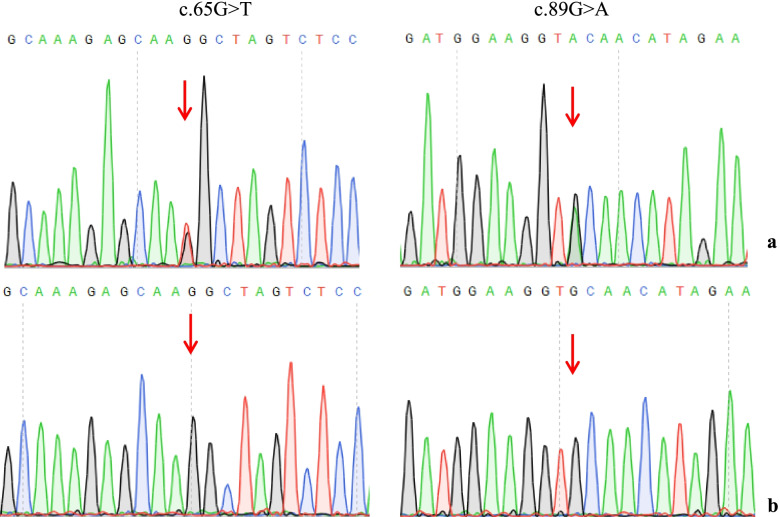
Direct sequencing results of *KCNJ1*. Compound mutations c.65G > T and c.89G > A in *KCNJ1* gene (NM_153767) in the proband (**a**), originated form his father and mother. The sites as normal control were shown in (**b**)

Two heterozygous mutation sites of *KCNJ1* gene (NM_153767) were found in the proband by NGS and convinced by PCR and Sanger sequencing, which were originated from his father and mother (Fig. 2). Both mutations were located at exon 4. One site c.89G > A (p.C30Y) was a known pathogenic mutation of *KCNJ1* [5]. The other one c.65G > T (p.R22M) was a novel missense mutation, leading to the arginine being substituted by methionine at codon 22. This missense mutation was absent from the HGMD, Single Nucleotide Polymorphism (SNP) database (dbSNP), 1000 Genomes Project (TGP) database and ClinVar database. Additionally, to our knowledge, this mutation has not been described in the Universal Mutation Database *KCNJ1* database or reported in any published literature. This missense mutation was predicted to be deleterious by SIFT, Polyphen-2 and MutationTaster (Table 1), which might be responsible for this family. Based on the ACMG guidelines, the c.65G > T (p.R22M) variant of *KCNJ1* was predicted to be Variant of Uncertain Significance (VUS)(PM2 + PP2 + PP3). Clinical reports indicated that the c.89G > A (p.C30Y) mutation was pathogenic [6].

### Functional analysis

**Fig. 3:**
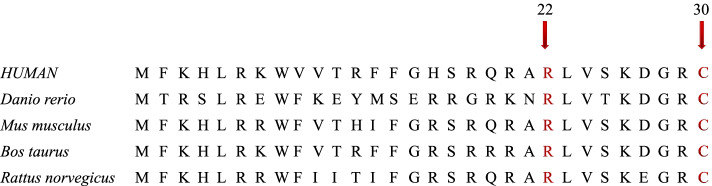
Phylogenetic comparison of protein encoded by *KCNJ 1* across species

**Fig. 4:**
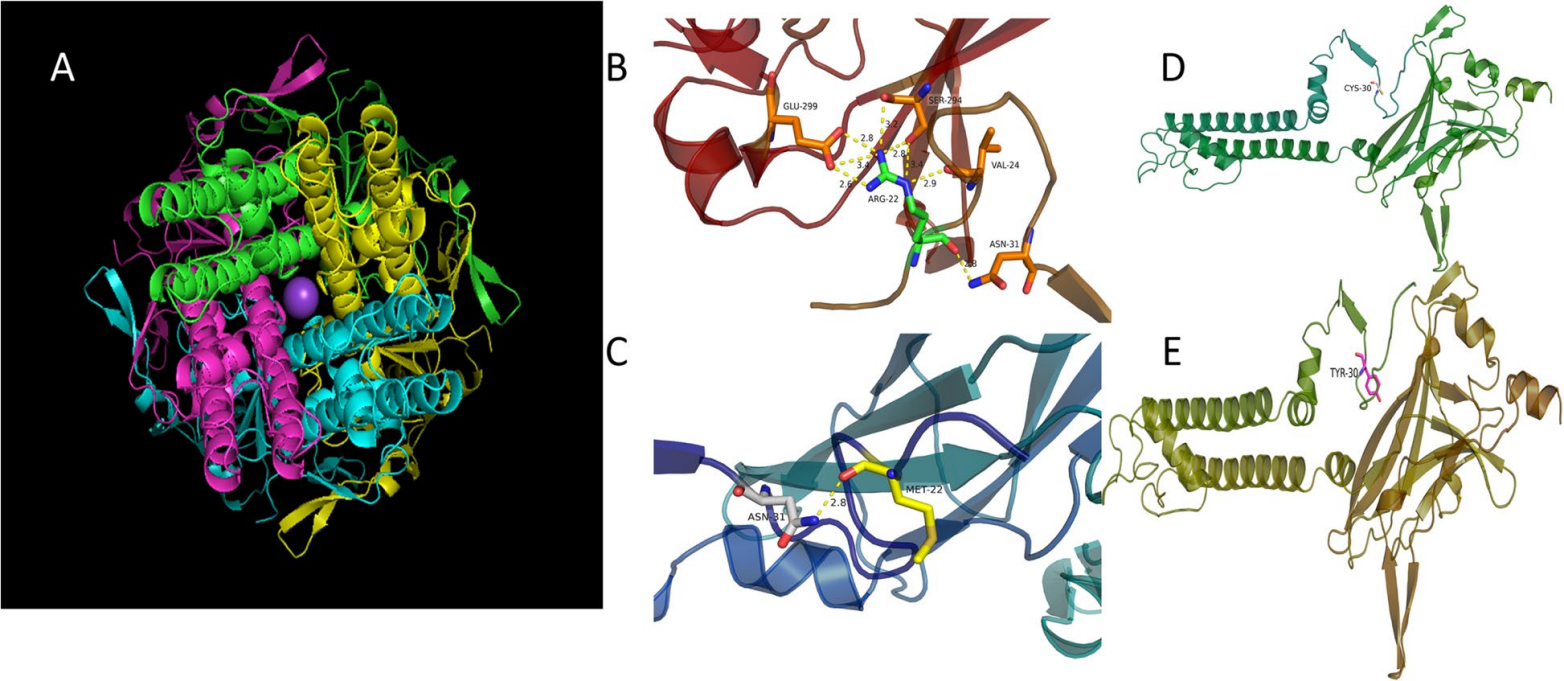
Homology modeling of wild-type and mutant *KCNJ 1* variants. **A** Modeled structure of the ROMK protein; **B** Neighboring residues of Arg22 in the wild type of *KCNJ1*. Arg22 is shown in green; **C** Neighboring residues of Met22 in mutant *KCNJ 1*. Met22 is shown in yellow. **D** Neighboring residues of Cys30 in the wild type of *KCNJ1*. Cys30 is shown in white; **E** Neighboring residues of Tyr30 in mutant *KCNJ1*. Tyr30 is shown in magenta. Predicted H bonds are indicated by yellow dashed lines

**Fig. 5:**
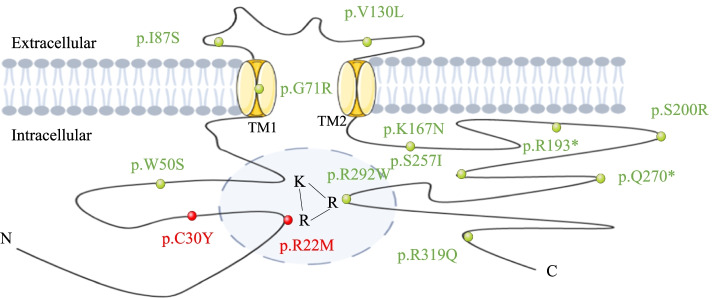
Structural model of ROMK1 channel protein and the position of the two variants identified in this study shown in red, the variants of ROMK1 detected in ref. [7] in green

An alignment of ROMK revealed that p.R22 and p.C30 are highly conserved among many different species (Fig. 3). Homology modelings of wild-type and mutant *KCNJ1* variants are shown in Fig. 4 and Fig. 5. The mutation results in a change from a basic amino acid (Arg) to a neutral amino acid (Met) at residue 22. The predicted model showed that the hydrogen bonds decreased. p.R22M could change the conformation of an arginine/lysine/arginine triad (KRR) and produce steric clashes with spatially adjacent residues, causing structural destabilization. The p.C30Y mutation caused a side-chain change of residue, which may affect protein structure and function.

## Discussion

In the present study, the proband, a ten-year old boy primarily incidentally detected with bilateral nephrolithiasis was found with compound heterozygous mutations of *KCNJ1* gene by NGS. *KCNJ1* gene is one of the five types of genes involved in the etiology of Bartter syndrome type II (BS II) which is a group of rare tubulopathies. Patients with different type of BS present with overlapping clinical phenotypes as polyuria, polydipsia, volume contraction, muscle weakness and growth retardation induced from hypokalaemia, hyperreninism and hyperaldosteronism. According to the onset and severity of BS, it can be grouped into three types: the hypocalciuric-hypomagnesemic variant described by Gitelman et al., the classic syndrome originally described by Bartter et al., and the antenatal hypercalciuric variant associated with severe systemic manifestations classical type [8]. *KCNJ1* gene encodes the apical potassium inwardly-rectifying channel (ROMK) in the thick ascending limb of the Henle’s loop (TALH) in the distal nephron to ensure adequate luminal potassium available for the efficient function of the Na-K-2Cl cotransporter which is involved in salt reabsorption. Effective chloride reabsorption in the TALH prevents renal salt wasting and is an essential mechanism to maintain tubular concentrating capability. Loss-of-functional mutations in the *KCNJ1* gene cause antenatal/neonatal BS II in autosomal recessive pattern [9].

In this study, two mutations of *KCNJ1* c.89G > A (p.C30Y) and c.65G > T (p.R22M) were detected in the proband. The distribution of the identified variations in *KCNJ1* is shown in Fig. 5. ROMK is responsible for K^+^ secretion and control of NaCl absorption in the kidney. The channel is gated by intracellular pH in the neutral range and reach half-maximal activation at a pH of 6.8. The gating is driven by the protonation of lysine within KRR [10], which is assembled by amino acid residues at positions 22, 61, and 292 in the transmembrane region [7]. Structural disturbance of KRR shifts the pKa of the lysine residue away from the neutral pH range and leads to channel inactivation. The predicted model of p.R22M revealed that the distance to Arg292 (11.8 Å) and Lys61 (30.3 Å) changed into 13.1 Å, and 31.2 Å, respectively, when the mutation replaced Arg22 with Met22. The p.R22M mutation could disrupt the conformation of KRR and produce steric clashes with spatially adjacent residues, causing structural destabilization. A three-dimensional structural analysis also revealed that Arg22 formed H bonds with Glu299, Ser294, and Val24. When the mutation replaced Arg22 with Met22, these H bonds were destroyed. In summary, the p.R22M mutation was able to influence KRR in two ways, either shifting the pKa of the lysine residue off the neutral pH range or influencing the tertiary geometry to further change the integrity of the structure and function of ROMK. The p.C30Y mutation caused a side-chain change of residue, which may affect protein structure and function. Clinical reports indicated that the p.C30Y mutation was pathogenic. Through the symptoms and genetic test, the proband was confirmed with type II Bartter syndrome (BS-II).

The Phenotype in most of patients with BS II can begin in utero with marked fetal polyuria presenting polyhydramnios from 24 weeks of gestation and premature delivery. During neonatal period, patients may have life-threatening volume depletion caused by severe renal salt wasting or failure to thrive. During childhood, other secondary symptoms including developmental retardation, fever, vomitting, occasional diarrhea may present. All the symptoms resulted from metabolic alkalosis, hyposthenuria, hyperreninaemic, hyperaldosteronism which was stimulated by elevated plasma concentration of prostaglandin E2 (PGE2). The basic deficiency of antenatal BS is the malfunction of mTAL chloride transport, which involves an interaction among the apical Na-K-2Cl cotransporter (*NKCC2*), the luminal ATP-sensitive potassium channel ROMK, the basolateral chloride channel (ClC), a basolateral K-CL cotransporter and the Na-K-ATPase. Therefore, any gene encoding or involving in these channels or transporters will result in defective chloride transport. *NKCC2*, *KCNJ1*, *CLCNKB* for chloride channel and *BSND* gene encoding barttin, a subunit for ClC-Ka and ClC-Kb have been confirmed with antenatal BS [11, 12]. Rare disease shall also be differentiated from Rabson-Medndenhall syndrome caused by *INSR* [13].

The other equally important feature in antenatal BS is hypercalciuria. Continuous loss of calcium results in nephrocalcinosis, nephrolithiasis and osteopenia [14, 15], usually medullary nephrocalcinosis is seen [16, 17]. Hypercalciuria and associated nephrocalcinosis are present in approximately 85% of infants with this neonatal BS [18]. The prevalence of nephrolithiasis is high, but the prevalence secondary to BS is not known very well, and may be lower than the prevalence of nephrocalcinosis. Both nephrocalcinosis and nephrolithiasis share a well-recognized heritability [19, 20], and around 15% of the patients were detected with causative genes [4]. Although low plasma potassium concentration, secondary low urinary citrate, tubulointerstitial damage, chloride deficiency, and increased intracellular chloride activity were also suggested to contribute to the hypercalciuria, the exact pathogenesis of nephrocalcinosis or nephrolithiasis in BS remains unclear [21]. Renal function is generally well preserved. In the present study, GFR of the proband was lower than the normal population. According to the ten-year outcome study by Puricelli E et al. [22], 25% of the patients with type I or type II BS had GFR lower than the normal range, which may be resulted from nephrocalcinosis. More than 30 genes have been reported to be with the etiology of nephrolithiasis [4]. Two-thirds of the genes currently known to be associated with nephrolithiasis coding for membrane proteins or enzymes involved in renal tubular transport [23]. The TALH and connecting tubules (CNT) have a central role in maintenance of fluid, electrolytes and acid-base homeostasis. Therefore, mutations of genes involved in TALH and CNT function can result in phenotypically severe disease. 14 of all genes are of paramount importance accounting for 15% of nephrolithiasis or nephrocalcinosis [24]. Recessive causes were more frequent among children, whereas dominant disease occurred more abundantly in adults. Therefore, NGS panel including genes involved in functions of TALH, connecting tubules, systemic disorders such as chromic hypercalcemia from vitamin D, primary hyperoxaluria, ARPT deficiency, distal renal tubular acidosis, Dent’s disease, cystinuria and family hypomagnesemia with hypercalciuria shall be applied [25, 26]. In this study, 248 genes associated with hereditary kidney diseases were all included in the panel, and no other suspicious gene mutations were found except *KCNJ1* gene.

*KCNJ1* gene mutation associated antenatal BS is phenotypically distinct from the other disease because of prominent polyhydramnios with preterm delivery together with discontinuous fatigue, still phenotypic variability presents in patients with *KCNJ1* mutation and absence of enough recognition for this type of disease may exist. The patient in the present study was not gotten accurate diagnosis until he was ten-years old and incidentally found bilateral nephrolithiasis, although he had the previous infant history with polyhydramnios and preterm delivery, and the intermittent cramps, fatigue and muscle weakness during childhood.

There are other causes which could also induce either of these symptoms. The clinicians or parents may ignore the real etiology beneath the manifestations and the clinical misdiagnosis of BS was nearly 25%, especially in developing countries [27]. Also the onset of BS type may be late. One adult male patient initially presented with an incidental finding of nephrocalcinosis was diagnosed as a late-onset BS due to detection of a homozygous *KCNJ1* missense mutation [28].

Our case showed that the presentations in patients with BS may not be unusual, and specific disorders within the spectrum of BS or nephrolithiasis may not easily be diagnosed or differentiated by rigorous clinical manifestations. Genetic test, especially NGS is a very efficient tool to distinguish specific disorder from multiple confusing spectrums.

## Data Availability

Datasets used in this article are available from corresponding author on reasonable request.
